# Porous intermetallic Ni_2_XAl (X = Ti or Zr) nanoparticles prepared from oxide precursors†

**DOI:** 10.1039/d1na00047k

**Published:** 2021-02-22

**Authors:** Yasukazu Kobayashi, Shohei Tada, Ryuji Kikuchi

**Affiliations:** Interdisciplinary Research Center for Catalytic Chemistry, National Institute of Advanced Industrial Science and Technology (AIST) 1-1-1 Higashi Tsukuba Ibaraki 305-8565 Japan yasu-kobayashi@aist.go.jp; Department of Materials Science and Engineering, Ibaraki University 4-12-1 Nakanarusawacho Hitachi Ibaraki 316-8511 Japan; Department of Chemical System Engineering, The University of Tokyo 7-3-1 Hongo Bunkyo-ku Tokyo 113-8656 Japan

## Abstract

Porous intermetallic Ni_2_XAl (X = Ti or Zr) nanoparticles with small crystallite sizes (24–34 nm) and high Brunauer–Emmett–Teller (BET) surface areas (10–71 m^2^ g^−1^) were prepared from oxide precursors by a chemical route. CaH_2_ acted as a template to form the porous morphologies and assisted the reduction.

## Abstract 2

Chemical synthesis of porous intermetallic Ni_2_XAl (X = Ti or Zr) nanoparticles with small crystal size (24–34 nm) and high surface area (10–71 m^2^ g^−1^).

## Graphical abstract

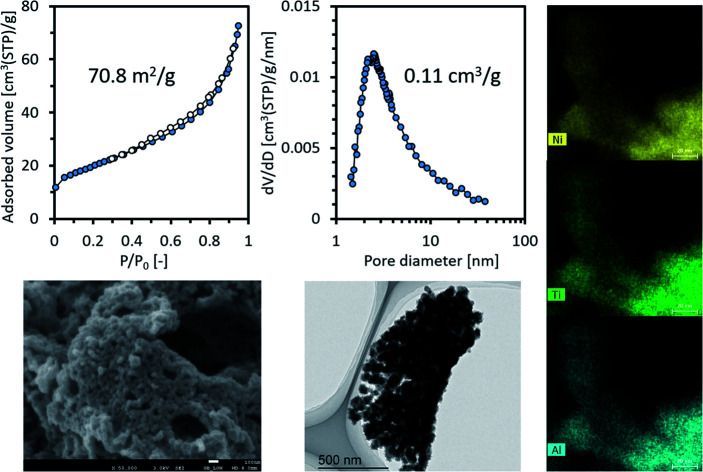

Heusler intermetallic compounds have attracted interest because of their applications in magnets, semiconductors, and thermoelectric materials.^1–3^ They are denoted as XYZ (half-Heusler structure) or X_2_YZ (full-Heusler structure), where X and Y are transition metals, and Z is a p-block metal. Some Heusler compounds such as Co_2_MnGe and Ni_2_TiAl have shown catalytic activities in hydrogenation and oxidation.^4–6^ Their catalytic activities can be tuned by substitution (*e.g.*, X_2_YZ_1−*x*_Z′_*x*_), allowing for fine adjustments of the electronic state (ligand effect) and atomic arrangement (ensemble effect) on the surface where heterogeneous catalytic reactions occur. However, difficulty in obtaining Heusler intermetallic powders with high surface areas hinders their practical application as catalysts. Commonly, preparing high-surface-area intermetallics, including early transition metals such as La, Y, Ti, and Zr, is challenging because of the strong oxygen affinities of these metals.^7–9^ Therefore, they have been prepared by physical approaches in oxygen- and moisture-free conditions. Zr_0.5_Hf_0.5_CoSb_0.8_Sn_0.2_,^10^ Hf_0.75_Zr_0.25_NiSn_0.99_Sb_0.01_,^11^ and MCo_1−*x*_Fe_*x*_Sn_*y*_Sb_1−*y*_ (M = Ti, Zr, and Hf)^12^ have been prepared by mechanical ball milling. Vapor deposition-based approaches have been used to prepare Fe_3_Si^13^ and Co_3_Si.^14^ However, these methods are not practical for applications,^15–17^ whereas the chemical approach is more scalable but challenging. Chemical preparation of some Heusler nanoparticles, such as Fe_3_Si^18^ and Co_2_FeAl,^19–23^ has been reported, but chemically prepared Heusler nanoparticles involving early transition metals have not been reported. Besides, although multicomponent alloys with early transition metals have been studied, such as high-entropy PtPdRhRuCe for ammonia oxidation^24^ and high-entropy CoFeLaNiPt for electrocatalytic water splitting,^25^ it is necessary to develop simple and versatile techniques for the preparation of nano-sized multicomponent alloys involving hard-to-reduce metals.

Previously, we have reported the preparation of high-surface-area intermetallic nanoparticles, Ni_3_Al (13–27 m^2^ g^−1^)^26,27^ and NiAl (94–114 m^2^ g^−1^),^27,28^ by a chemical route. NiAl oxide precursors, NiO and NiAl_2_O_4_, were reduced to form porous intermetallic nanostructures in a molten LiCl–CaH_2_ system. In the molten LiCl at 600 °C, CaH_2_ works as a reducing as well as dehydration agent. Although pure Al tends to react with oxygen, molten LiCl prevents oxide formation and promotes the formation of NiAl and Ni_3_Al at 600 °C. The obtained intermetallic nanoparticles had high surface areas attributed to porous structures formed by CaO and CaH_2_, which acted as a template. The proposed preparation method was then applied to various intermetallic nanoparticles, such as YNi_2_Si_2_, LaNi_2_Si_2_,^29^ Pt_2_Y,^30^ and NiZn.^31^ In this study, we prepared high-surface-area Heusler nanoparticles comprising early transition metals. Ni_2_TiAl was selected because of its catalytic applications in steam reforming of methanol,^6^ whereas Ni_2_ZrAl was prepared to demonstrate the versatility of the method. This is the first report on the chemical preparation of Heusler nanoparticles involving early transition metals. Finally, the prepared nanoparticles were used for CO_2_ activation to evaluate their catalytic performances.

Intermetallic Ni_2_XAl (X = Ti or Zr) nanoparticles were prepared by reducing oxide precursors in a LiCl–CaH_2_ mixture at 600 °C.^26–31^ First, Ni(NO_3_)_2_·6H_2_O, X material (TiCl_4_ or ZrO(NO_3_)_2_·2H_2_O), Al(NO_3_)_3_·9H_2_O, and glycine were dissolved in distilled water in a molar ratio of Ni/X/Al/glycine = 2/1/1/4.8. The solution was then dried at 110 °C overnight, and the dried powder was finally heated at 500 °C in the air for 2 h to obtain the oxide precursor Ni_2_TiAl(Pre) or Ni_2_ZrAl(Pre). The oxide precursor CaH_2_ and LiCl were next mixed in a mortar in a weight ratio of precursor/CaH_2_/LiCl = 0.2/0.4/0.2. The mixed powder was then loaded in a stainless-steel reactor and heated at 600 °C for 2 h under Ar. Finally, the treated precursor was crushed in a mortar and rinsed with 0.1 M NH_4_Cl aqueous solution and distilled water, and the final powder, Ni_2_TiAl(RDT) or Ni_2_ZrAl(RDT), was obtained. Characterization and catalytic tests are described in ESI.†

Fig. 1 shows the X-ray diffraction (XRD) patterns of Ni_2_TiAl(Pre) and Ni_2_TiAl(RDT). For Ni_2_TiAl(Pre), the peaks were assigned to NiO and Al_2_O_3_. Titanium-based oxides were not observed. Next, the precursor was reduced by the chemical approach, where the oxide precursor was reduced in a LiCl–CaH_2_ system at 600 °C. For the reduced powder of Ni_2_TiAl(RDT), the XRD peaks were mostly assigned to the intermetallic Ni_2_TiAl phase. The crystallite size of Ni_2_TiAl was estimated as 24 nm from the Scherrer equation (Table S1†).

**Fig. 1:**
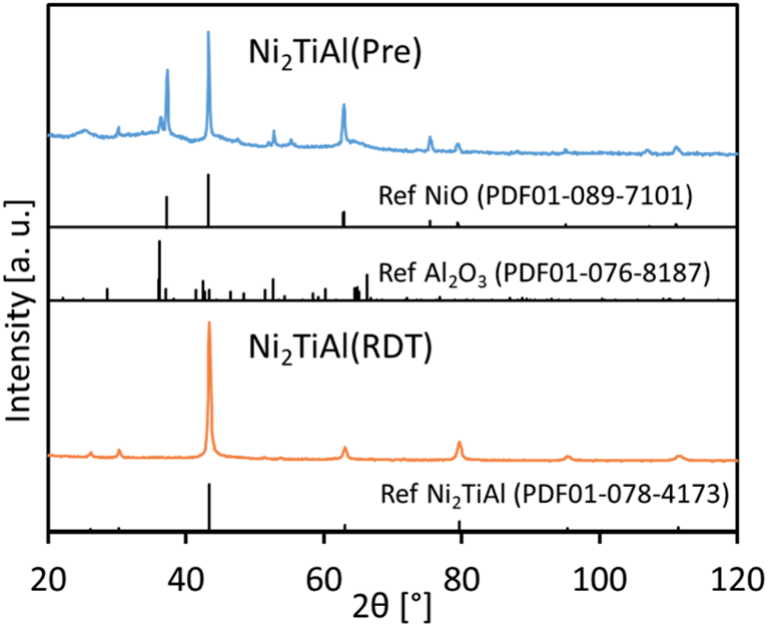
XRD patterns of an oxide precursor of Ni_2_TiAl(Pre) and the reduced sample of Ni_2_TiAl(RDT).

The morphology of Ni_2_TiAl(RDT) was then investigated by Scanning Electron Microscope (SEM) (Fig. 2 and S1–S3†) and (Scanning) Transmission Electron Microscope ((S)TEM) (Fig. 3 and S4–S8†) with energy-dispersive X-ray spectroscopy (EDS) analysis. Small particles of <100 nm interconnected to form porous structures. Moiré fringes indicate good crystallinity. Molar ratios of Ni, Ti, and Al in Ni_2_TiAl(RDT) calculated by SEM and TEM-EDS were consistent with the stoichiometric ratio of the intermetallic Ni_2_TiAl phase. On the elemental mappings, Ni, Ti, and Al are distributed evenly, and their positions are overlapped, indicating the homogeneity of the Ni_2_TiAl phase. Molar ratios of oxygen were relatively high in SEM and TEM-EDS, and the high-angle annular dark-field (HAADF)-STEM images show a high concentration of oxygen on the sample surface (Fig. 3, S7 and S8†). Because Ti and Al are readily oxidized in comparison with Ni, the sample surface was covered by titanium and aluminum oxides although they were not identified by XRD. Impurities such as Ca, Cl, and Li were not detected in Ni_2_TiAl(RDT), except for oxygen. Therefore, rinsing with NH_4_Cl removed these element-related species, such as CaO, CaH_2_, CaCl_2_, LiCl, and so on.

**Fig. 2:**
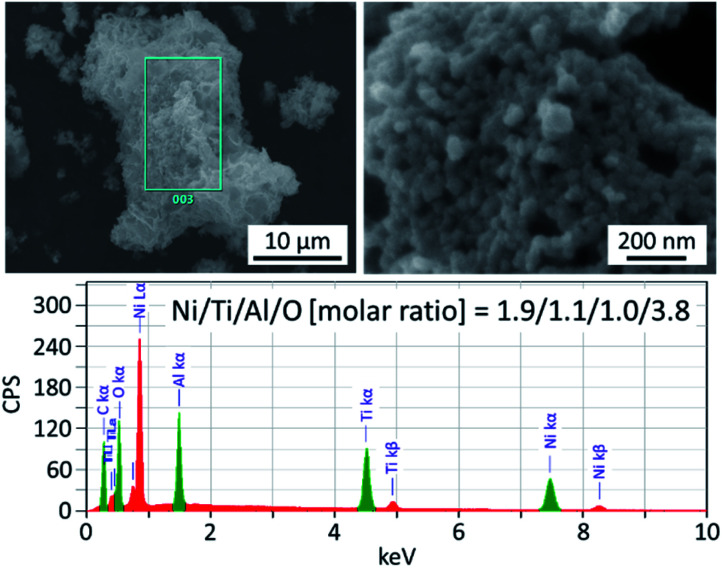
SEM images of Ni_2_TiAl(RDT) and its elemental analysis.

**Fig. 3:**
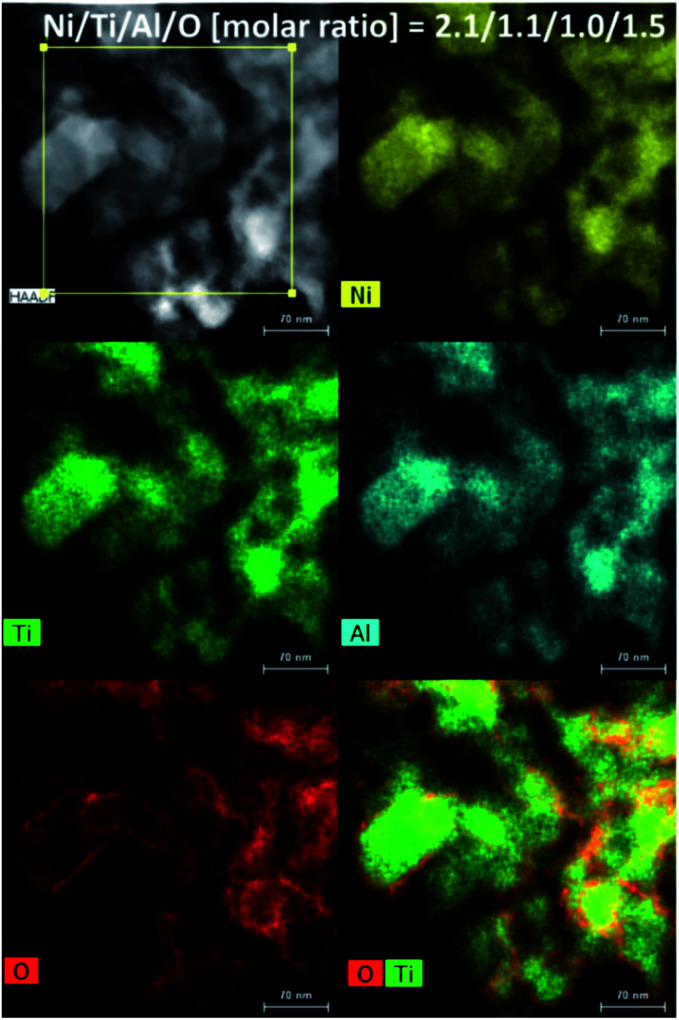
HAADF-STEM images of Ni_2_TiAl(RDT) and its elemental analysis.

The surface area and porosity of Ni_2_TiAl(RDT) were examined by nitrogen adsorption/desorption. Fig. 4 shows the isotherm curves and the corresponding pore size distribution. The physical values calculated from the curves are summarized in Table S1.† Ni_2_TiAl(RDT) showed large nitrogen adsorption indicated by a high BET surface area (71 m^2^ g^−1^). Small hysteresis was observed between the adsorption and desorption curves, and a porous structure was suggested by a Barrett–Joyner–Halenda (BJH) method with mesopore sizes of <10 nm. A similar pore size distribution was observed in our previous reports for mesoporous NiAl,^27,28^ indicating that calcium species such as CaH_2_ and CaO worked as a template in molten LiCl. Table S2† summarizes BET surface areas and preparation methods of reported nickel-based aluminides. The BET surface area of Ni_2_TiAl powder prepared by arc melting is very small (0.13 m^2^ g^−1^). Thus, the Ni_2_TiAl powder prepared in this study had more than two orders of magnitude BET surface area compared to that prepared by arc melting. In addition, the reported BET surface areas of nickel aluminides prepared by physical and chemical methods are 15–27 m^2^ g^−1^. Our method provided remarkably high BET surface areas because of the porous structures. Enhanced catalyst durability and stable activity in ordered nanoporous alloys have been suggested due to their superior mechanical strength.^32^ Thus the prepared porous intermetallic nanoparticles could be promising catalysts from the practical point of view.

**Fig. 4:**
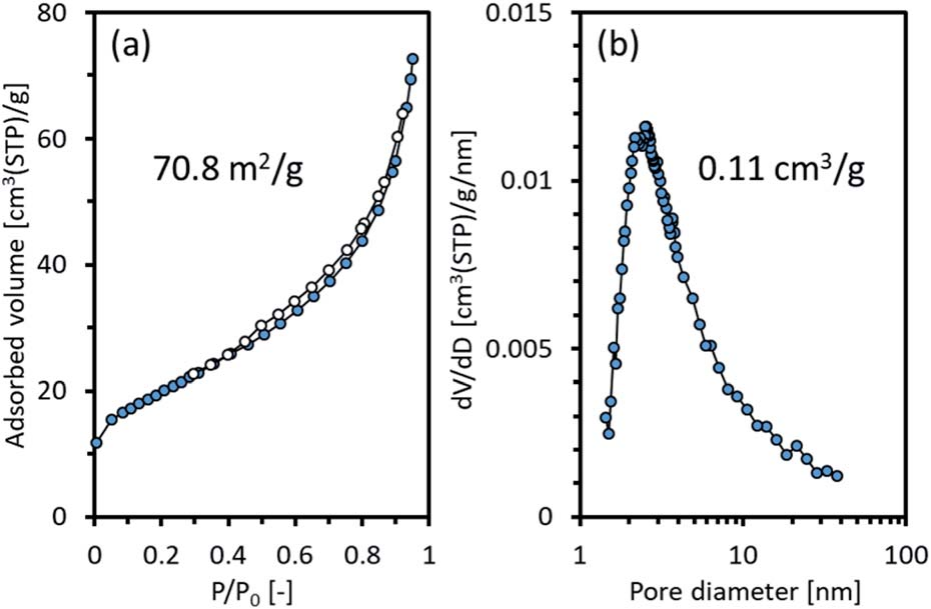
(a) Adsorption and desorption isotherms of nitrogen and (b) pore size distribution of Ni_2_TiAl(RDT).

Next, surface states of Ni_2_TiAl(RDT) and the Ar^+^-etched sample, denoted by Ni_2_TiAl(RDT)-etch, were analyzed by X-ray Photoelectron Spectroscopy (XPS) measurements. Fig. 5 shows the XPS spectra corresponding to Ni 2p_3/2_, Ti 2p_3/2_, Al 2p, and Ni 3p. In Fig. 5(a), the Ni 2p_3/2_ spectrum of Ni_2_TiAl(RDT) had a peak at 856.1 eV (ref. 33) assigned to Ni^3+^. It is noteworthy that the spectrum of Ni_2_TiAl(RDT)-etch had an intense peak of metallic Ni at 852.8 eV (ref. 34) (Fig. 5(c)), where an intense peak of metallic Ni was also observed at 66.1 eV.^35^ In Fig. 5(b), the Ti 2p_3/2_ spectrum of Ni_2_TiAl(RDT) had a peak at 459.6 eV of Ti^4+^, whereas the spectrum of Ni_2_TiAl(RDT)-etch was separated into four peaks: Ti^0^, Ti^2+^, Ti^3+^, and Ti^4+^.^36,37^ The results suggest that the etching treatment partially removed the titanium oxide layer. In Fig. 5(c), the Al 2p spectrum of Ni_2_TiAl(RDT) had a peak at 74.8 eV corresponding to AlO_*x*_,^38,39^ confirming the formation of the surface oxide layer. The spectrum of Ni_2_TiAl(RDT)-etch had a clear peak corresponding to metallic Al at 72.3 eV.^38,39^ These results for Ni_2_TiAl(RDT) indicate the presence of the surface oxide layer, consistent with the results of STEM observations. Importantly, the oxide layer was thin enough to be mostly removed by etching, and the existence of Ni–Ti–Al alloy, which could be an intermetallic Ni_2_TiAl phase, below the oxide layer was confirmed by XPS measurements. Thus, porous Ni_2_TiAl nanoparticles are suitable to be used in catalytic applications.

**Fig. 5:**
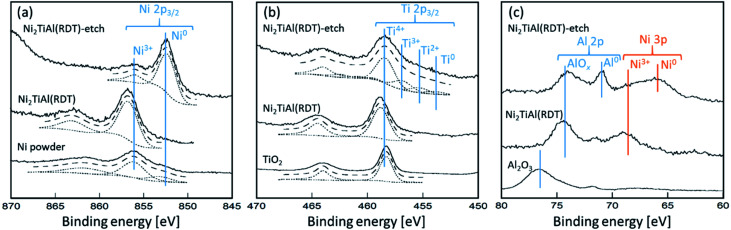
XPS spectra of (a) Ni 2p_3/2_, (b) Ti 2p_3/2_, and (c) Al 2p and Ni 3p for Ni_2_TiAl(RDT) and Ni_2_TiAl(RDT)-etch with references of Ni powder, TiO_2_, and Al_2_O_3_.

Next, Ni_2_ZrAl(RDT) was prepared and characterized similarly to Ni_2_TiAl(RDT) to demonstrate the versatility of the method (Fig. S9–S17 and Table S1†). The Ni_2_ZrAl phase was obtained with a crystallite size of 34.4 nm and a BET surface area of 10.3 m^2^ g^−1^. Impurities such as NiAl, Ni_10_Zr_7_, and NiZr were observed, resulting in the lower surface area of Ni_2_ZrAl(RDT) compared with that of Ni_2_TiAl(RDT).

Finally, the catalytic performances of the prepared intermetallic compounds were evaluated in CO_2_ activation. The results are shown in Fig. 6. To evaluate the intrinsic catalytic performances, we calculated the turnover frequency (TOF) (Tables S3 and S4†). The TOF for CO_2_ activation, TOF(CO_2_), was in the order of Ni_2_ZrAl(RDT) > NiAl > Ni_2_TiAl(RDT) at 400 °C, indicating that Ni_2_ZrAl(RDT) activated CO_2_ more than did NiAl. In a lower reaction temperature range, TOF(CO_2_) and TOF(CH_4_) were in the order of NiAl > Ni_2_ZrAl(RDT) > Ni_2_TiAl(RDT). These trends indicated that the substitution of half of Al with Ti and Zr in NiAl harmed the CO_2_ activation for methane formation. The order of apparent activation energies (*E*_a_) was given as Ni_2_ZrAl(RDT) (104 kJ mol^−1^) > NiAl (65 kJ mol^−1^) > Ni_2_TiAl(RDT) (61 kJ mol^−1^). Ni_2_ZrAl(RDT) gave a high Ea value close to 99 kJ mol^−1^ of the CeO_2_-supported Ni catalyst.^40^ On the other hand, NiAl and Ni_2_TiAl(RDT) showed low Ea values similar to 70 kJ mol^−1^ of the sponge Ni catalyst.^40^ Thus, the active sites on NiAl and Ni_2_TiAl(RDT) could be in the same state as that of sponge Ni to catalyze CO_2_ activation, suggesting that the non-supported metallic catalysts could decrease the energy barrier for CO_2_ activation.

**Fig. 6:**
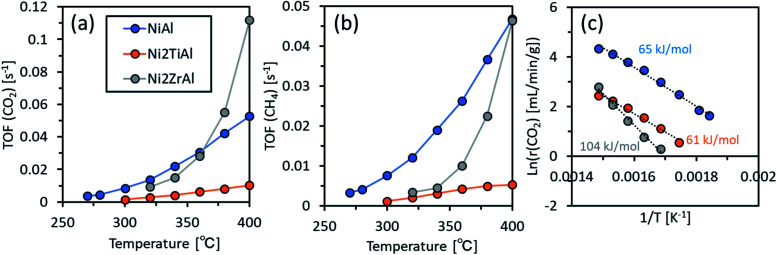
(a) TOF for CO_2_ activation and (b) TOF for CH_4_ production as functions of reaction temperature, and (c) the Arrhenius plots for NiAl, Ni_2_TiAl(RDT), and Ni_2_ZrAl(RDT).

## Conclusions

Porous intermetallic Ni_2_XAl (X = Ti or Zr) nanoparticles were prepared using CaH_2_ as a reducing agent in molten LiCl at 600 °C. The obtained BET surface areas were two orders of magnitude higher than those of powder samples prepared by arc melting. The versatility of our method allows for the preparation of porous intermetallic aluminide nanoparticles. The prepared Ni_2_ZrAl showed a higher TOF(CO_2_) at 400 °C than NiAl, whereas the Ni_2_TiAl activated CO_2_ with a lower activation energy than a reported supported catalyst. These findings suggested a potential advantageous application of the prepared nanoparticles in CO_2_ activation.

## Author contributions

YK carried out the experiments including synthesis and characterization. YK also conceptualized the project and supervised the research work. ST performed the experiments for XPS analysis and catalytic activity evaluation. RK discussed the results, helped to prepare the manuscript. All authors carried out the required revisions.

## Conflicts of interest

There are no conflicts to declare.

## Supplementary Material

NA-003-D1NA00047K-s001
