# Supplementary material for: Porous intermetallic Ni2XAl (X = Ti or Zr) nanoparticles prepared from oxide precursors
Source: Nanoscale Adv. 2021 Feb 22;3(7):1901–5. doi: 10.1039/d1na00047k (PMC9417867; doi:10.1039/d1na00047k)
Supplement: NA-003-D1NA00047K-s001 [file NA-003-D1NA00047K-s001.pdf]

Electronic Supplementary Material (ESI) for Nanoscale Advances.
This journal is © The Royal Society of Chemistry 2021

Electronic Supplementary Information

Porous intermetallic Ni_2XAl (X = Ti or Zr) nanoparticles prepared from oxide precursors

Yasukazu Kobayashi *[a], Shohei Tada [b], and Ryuji Kikuchi [c]

a Interdisciplinary Research Center for Catalytic Chemistry, National Institute of Advanced Industrial Science and Technology (AIST), 1-1-1 Higashi, Tsukuba, Ibaraki 305-8565, Japan.
b Department of Materials Science and Engineering, Ibaraki University, 4-12-1 Nakanarusawacho, Hitachi, Ibaraki 316-8511, Japan.
c Department of Chemical System Engineering, The University of Tokyo, 7-3-1 Hongo, Bunkyo-ku, Tokyo 113-8656, Japan.

**E-mail: yasu-kobayashi@aist.go.jp*

1. Experimental

1.1. Sample Characterization

The crystal structure was investigated by X-ray diffraction (XRD, SmartLab (3kW), Rigaku) with CuKα radiation at 40 kV and 45 mA. The porosity was examined by nitrogen adsorption/desorption at -196 °C (BELLSORP mini-II, Microtrac-BEL). The sample was pre-treated at 200 °C for 30 min under vacuum before the measurement. The pore size distribution was analyzed from the measured isotherms using the Barrett, Joyner, and Halenda (BJH) method. The morphology was observed by Scanning Electron Microscope (SEM, JSM-7800F, JEOL Ltd.) and (Scanning) Transmission Electron Microscope ((S)TEM, Tecnai Osiris, FEI) with energy-dispersive X-ray spectroscopy (EDS) for elemental analysis. Metallic/oxidation states of constituent elements in the final samples were confirmed by X-ray Photoelectron Spectroscopy (XPS, JPS-9010TR, JEOL Ltd.). The spectra were corrected by referencing the binding energy to carbon (C1s 284.6 eV). Commercial Nickel powder (Wako Pure Chem. Corp.), TiO_2 (JRC-TIO-15, Supplied by Catalysis Society of Japan) and Al_2O_3 (JRC-ALO-9, Supplied by Catalysis Society of Japan) were used for references. To remove the surface oxide layer of the sample, we used an Ar^+ etching ion gun (JEOL XP-HSIG, 600 V, 12 mA, 60 s).

1.2. Catalytic performance test

The catalytic performance for CO_2 activation was evaluated in a 6 mm (i.d.) fixed-bed tubular reactor at atmospheric pressure. The catalyst of 30 mg and quartz sand (Wako Chemicals Corp.) of 1 g was placed in the reactor and then pretreated at 500 °C for 1 h under a flow of 10%H_2/N_2 gas prior to each run. The feed gas consists of 10% CO_2, 40% H_2 and 50% He in a total flow rate of 50 mL/min. The gas composition at the reactor outlet was analyzed using non-dispersive infrared spectroscopy (NDIR) (VA-3111, HORIBA, Ltd.).

A porous intermetallic NiAl powder was also tested for comparison. It was prepared through the same preparation method as that of Ni_2TiAl(RDT) and Ni_2ZrAl(RDT). The crystal structure of these intermetallic compounds is all the same, which means that Ni_2TiAl and Ni_2ZrAl are obtainable by substituting a half of Al in a NiAl crystal structure with Ti or Zr. For all catalysts, the only products observed were CO, CH_4, and H_2O. Turnover frequencies (TOF) for CO_2 activation and CH_4 production were calculated to compare the intrinsic catalytic performances based on surface nickel site. The number of surface nickel sites was estimated from the BET surface area and atomic radii of the constituent elements in each catalyst (**Table S3**). With the

number of active sites, the TOF values for CO_2 activation (TOF(CO_2)) and CH_4 production (TOF(CH_4)) were then calculated from CO_2 reaction rate and CH_4 production rates, respectively. For the Arrhenius plots over NiAl, Ni_2TiAl(RDT) and Ni_2ZrAl(RDT), over the range of changed reaction temperature, good linearities were observed for all catalysts and the apparent activation energies (Ea) were obtained as listed in **Table S4.**

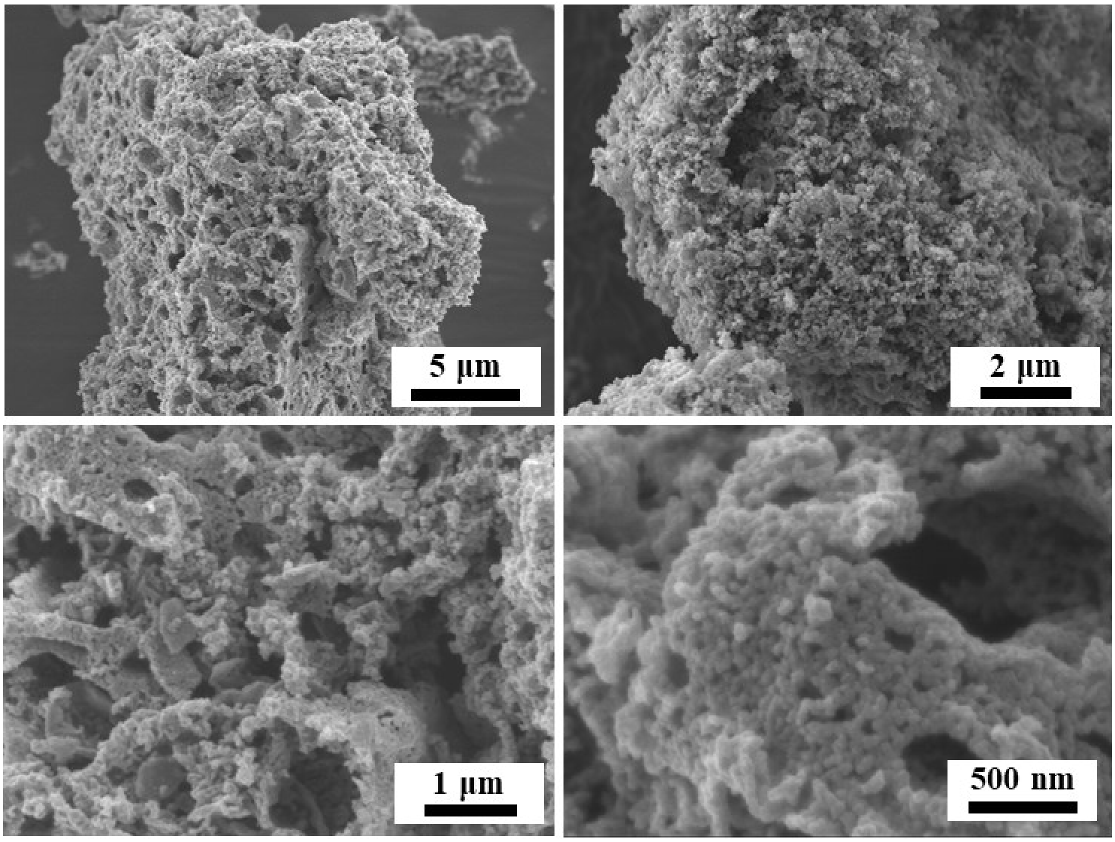

Fig. S1 SEM images of Ni_2TiAl(RDT).

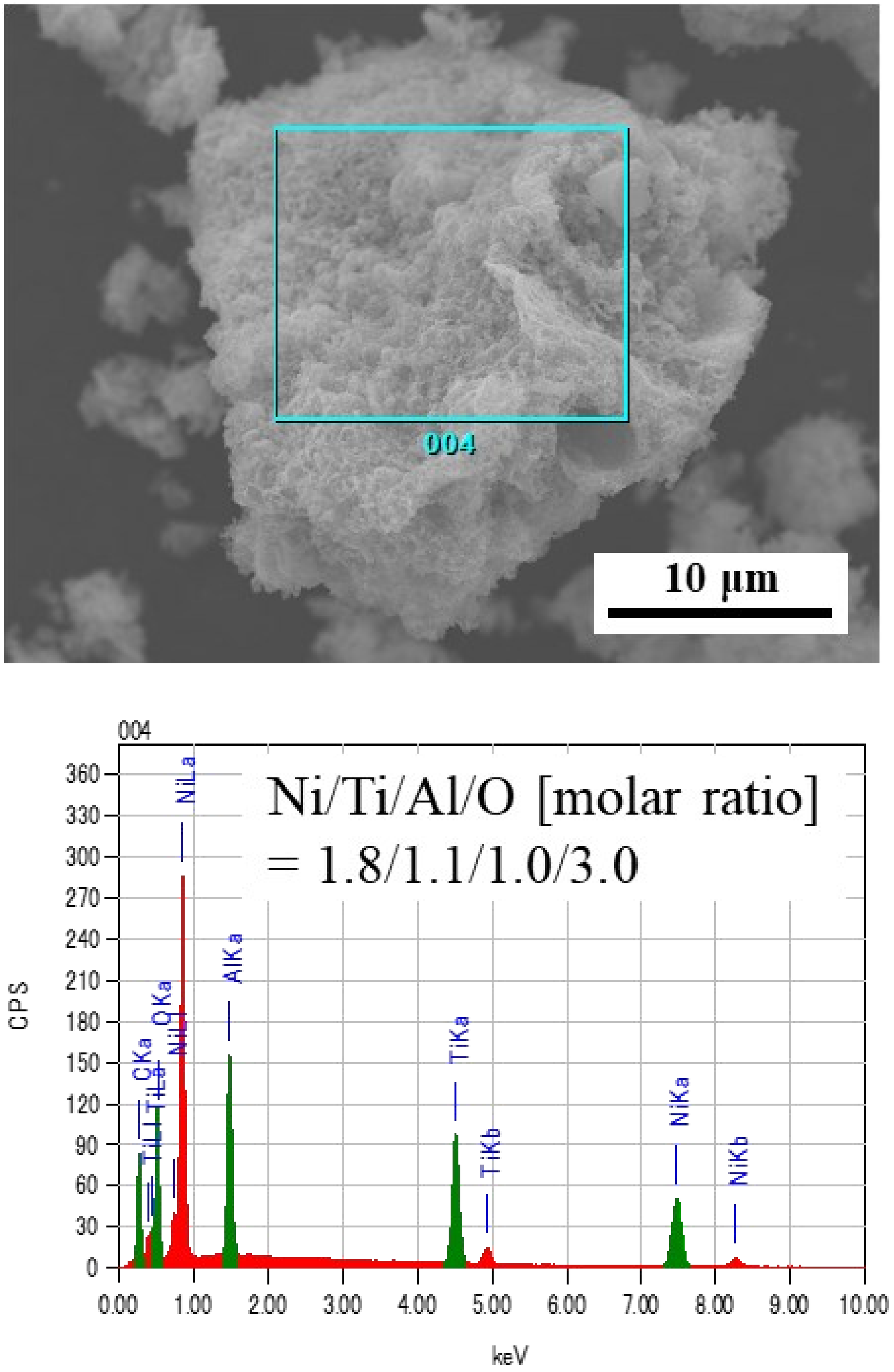

Fig. S2 A SEM image and the elemental analysis on Ni_2TiAl(RDT).

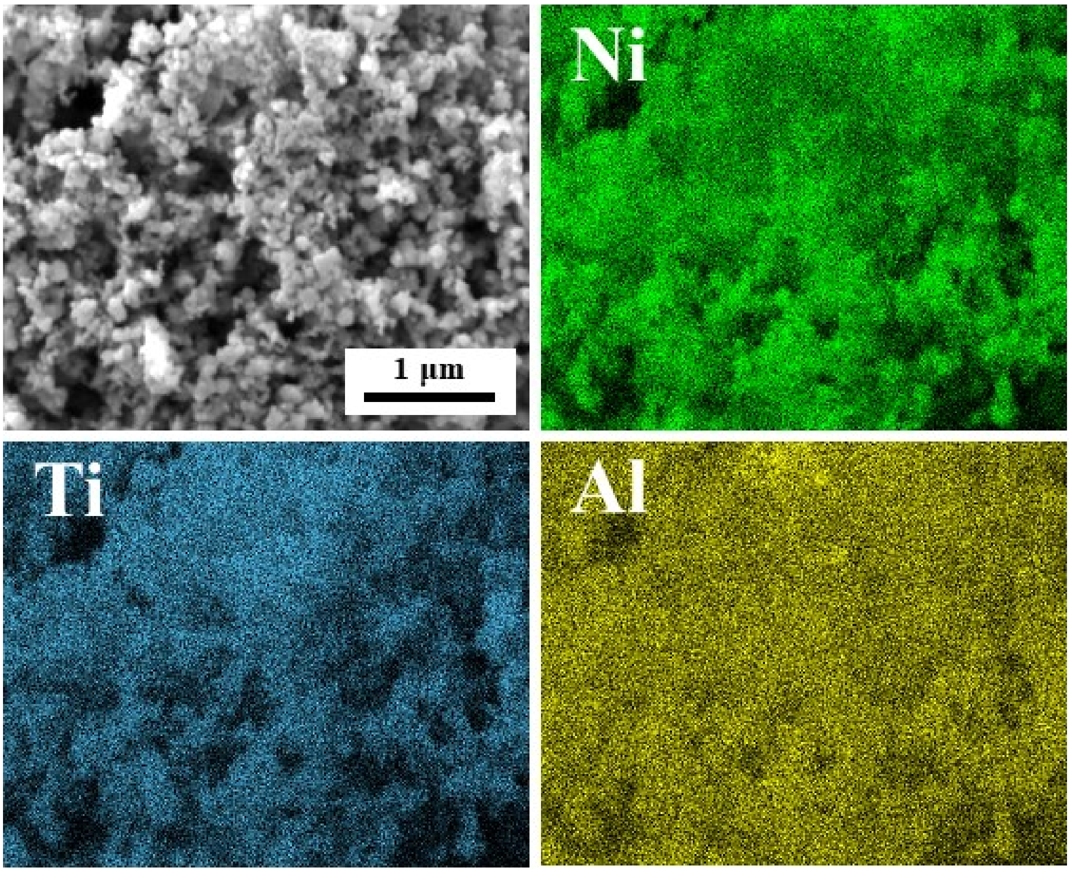

Fig. S3 A SEM image and the elemental mapping of Ni, Ti and Al on Ni_2TiAl(RDT).

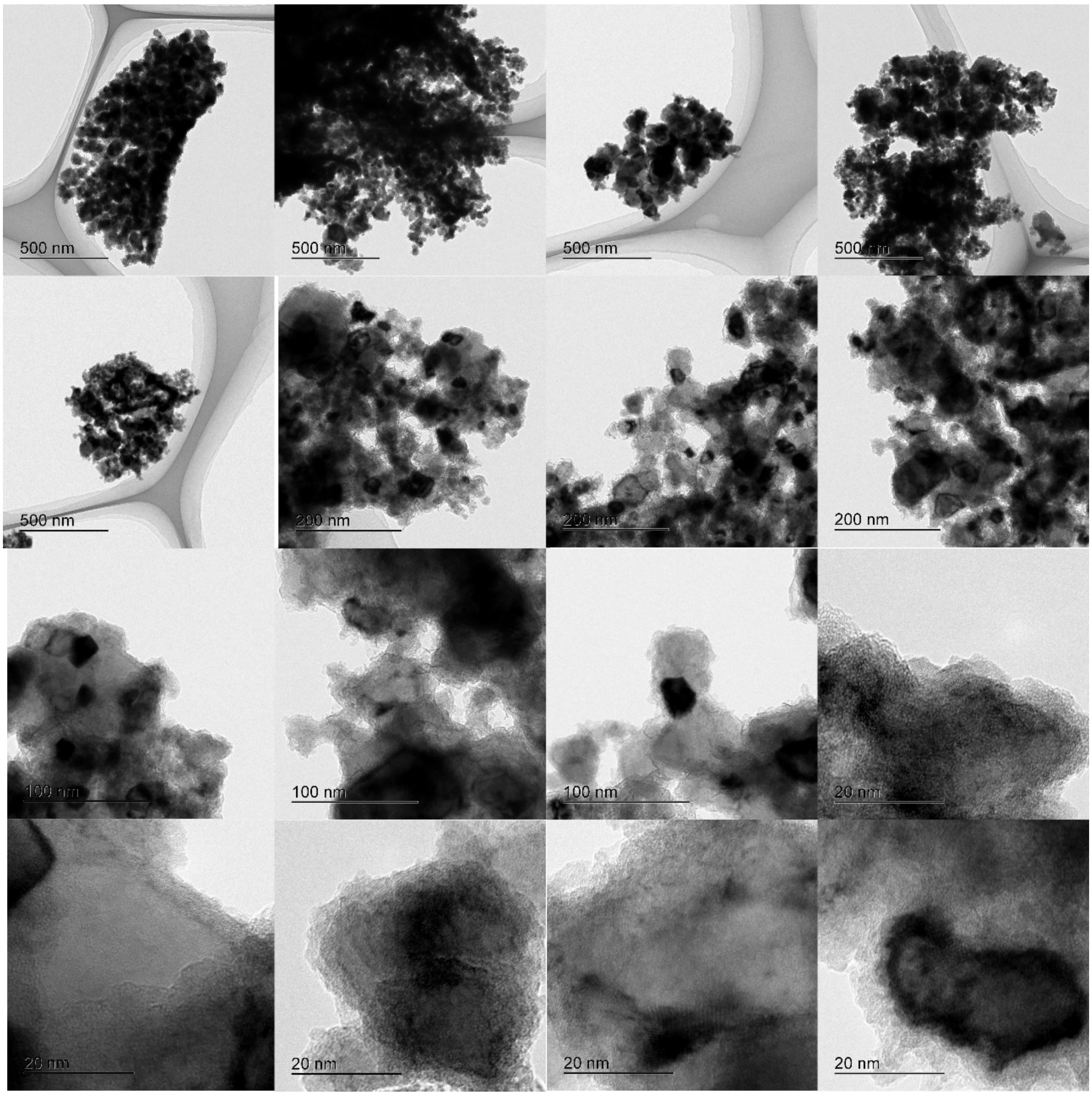

Fig. S4 TEM images of Ni_2TiAl(RDT).

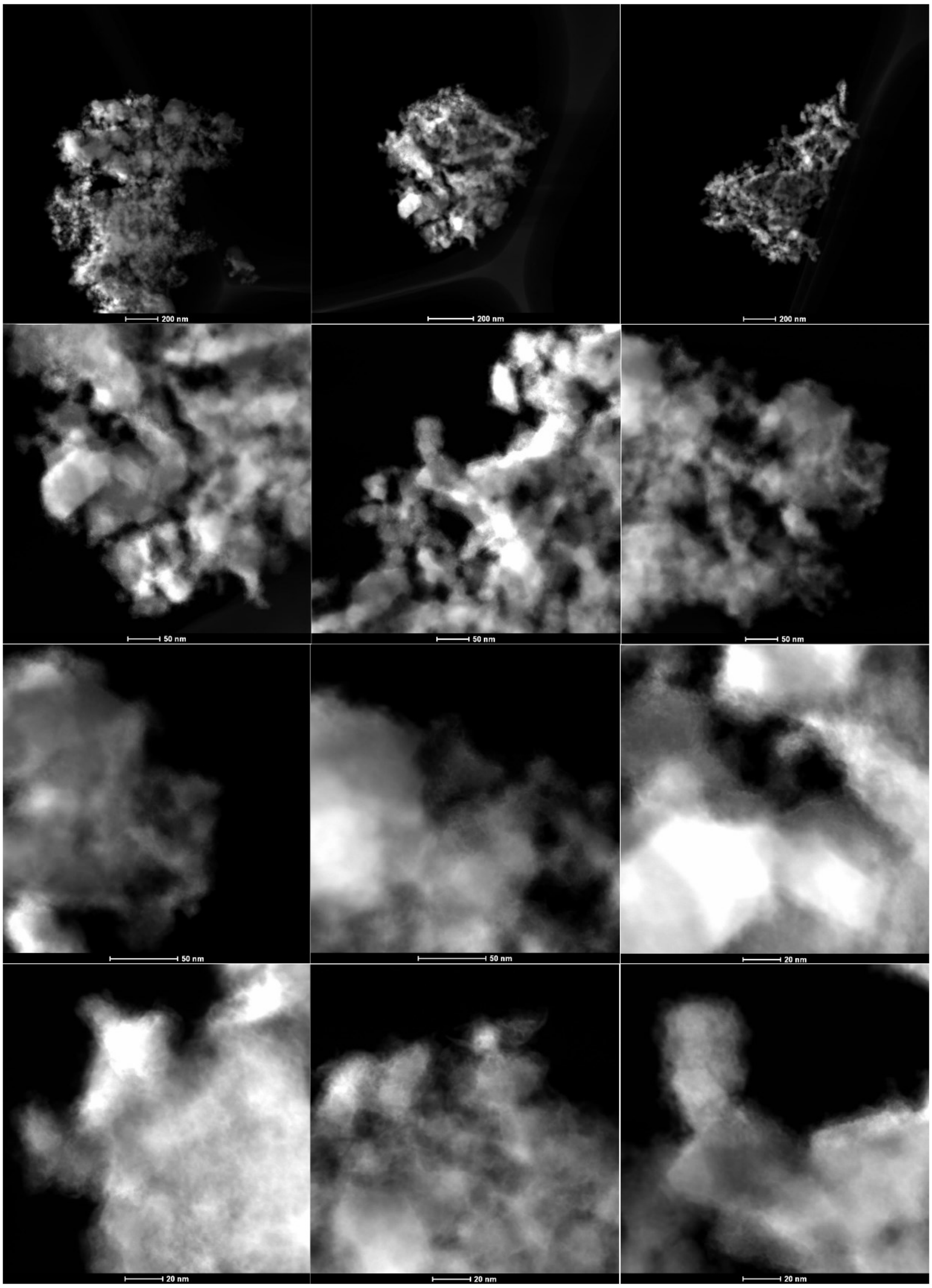

Fig. S5 HAADF-STEM images of Ni_2TiAl(RDT).

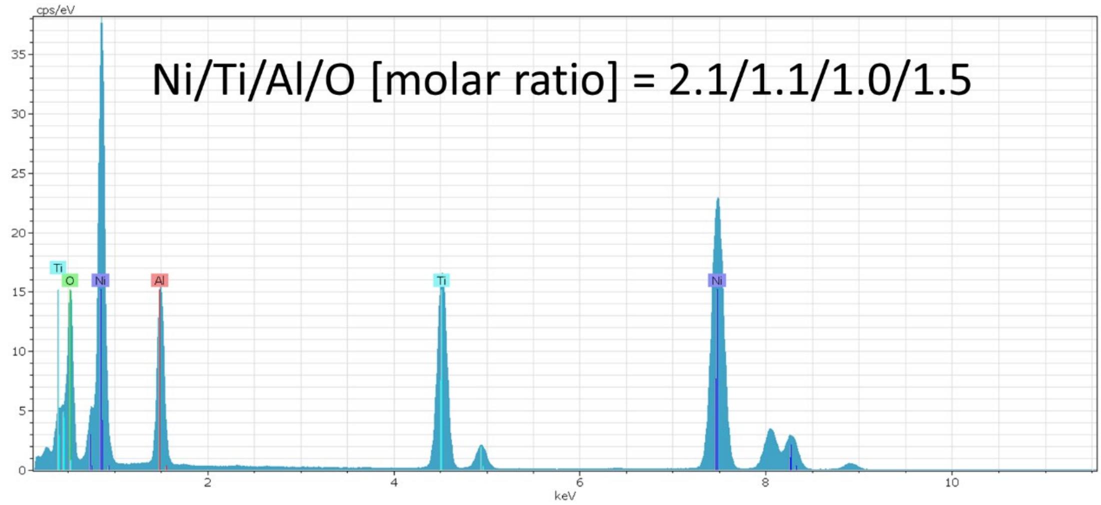

Fig. S6 Elemental analysis corresponding to the enclosed portion of a HAADF-STEM image in **Fig. 3** with molar ratios for Ni_2TiAl(RDT).

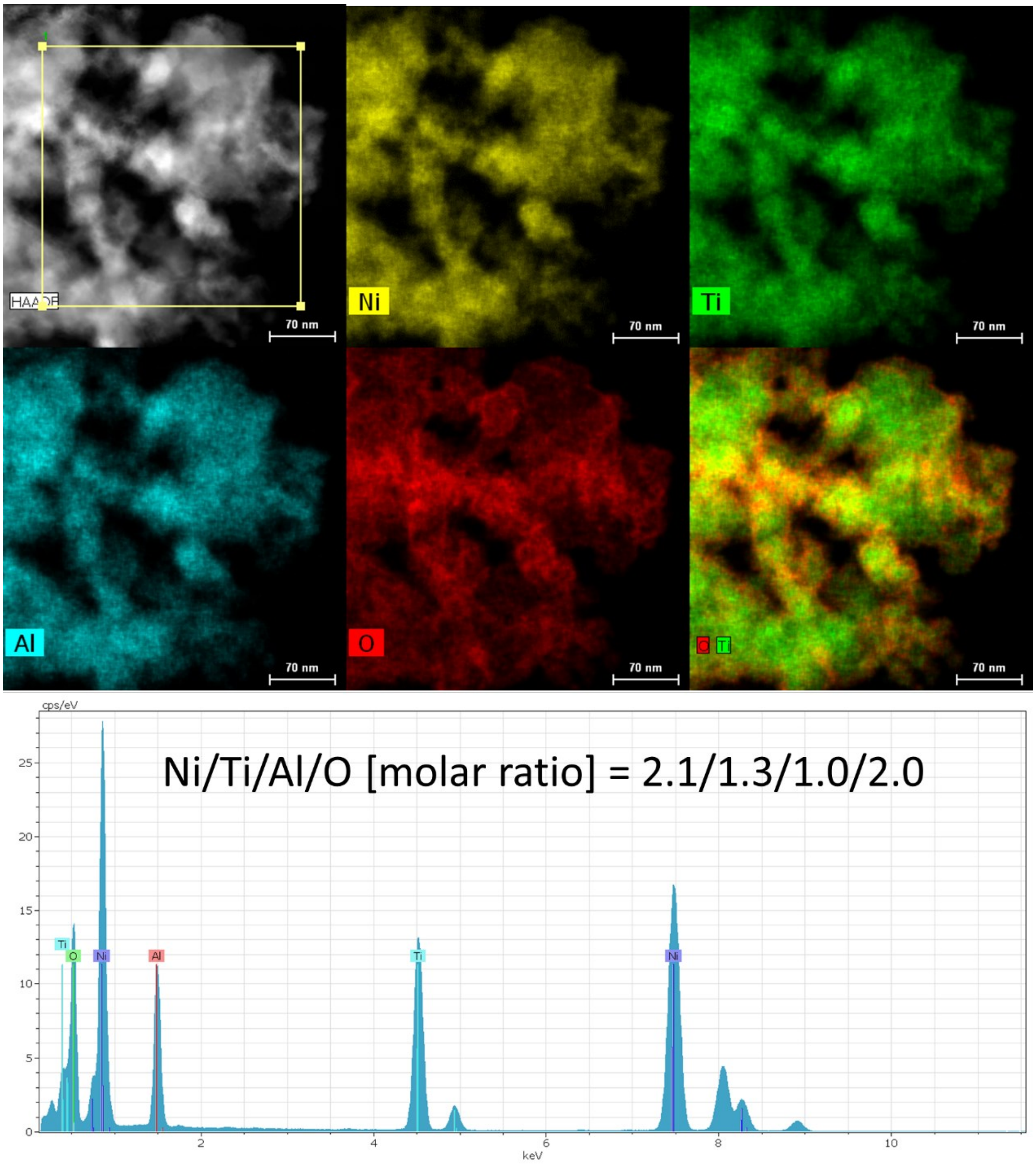

Fig. S7 HAADF-STEM images and elemental analysis of enclosed portion with molar ratios for Ni_2TiAl(RDT).

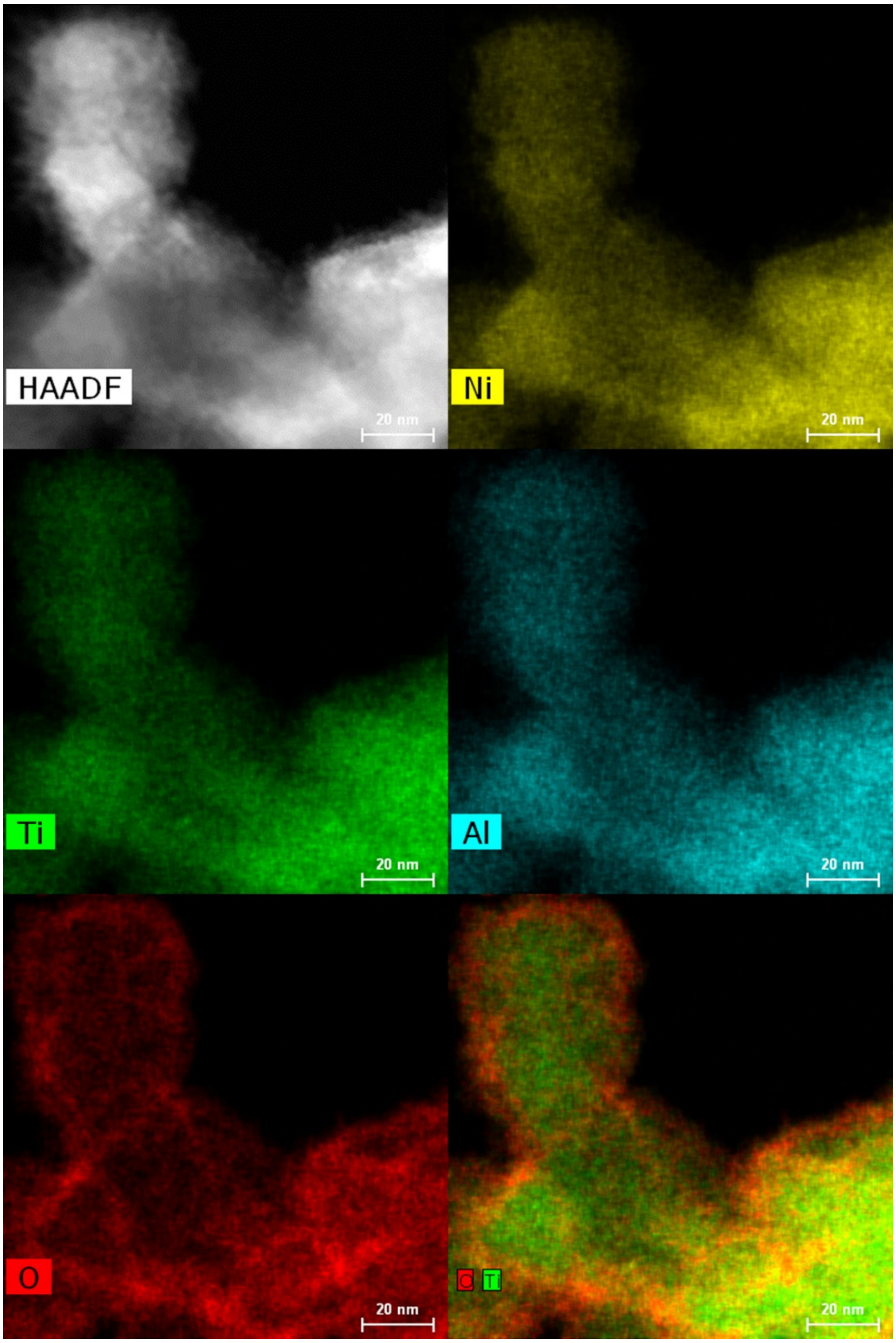

Fig. S8 HAADF-STEM images and elemental analysis for Ni_2TiAl(RDT).

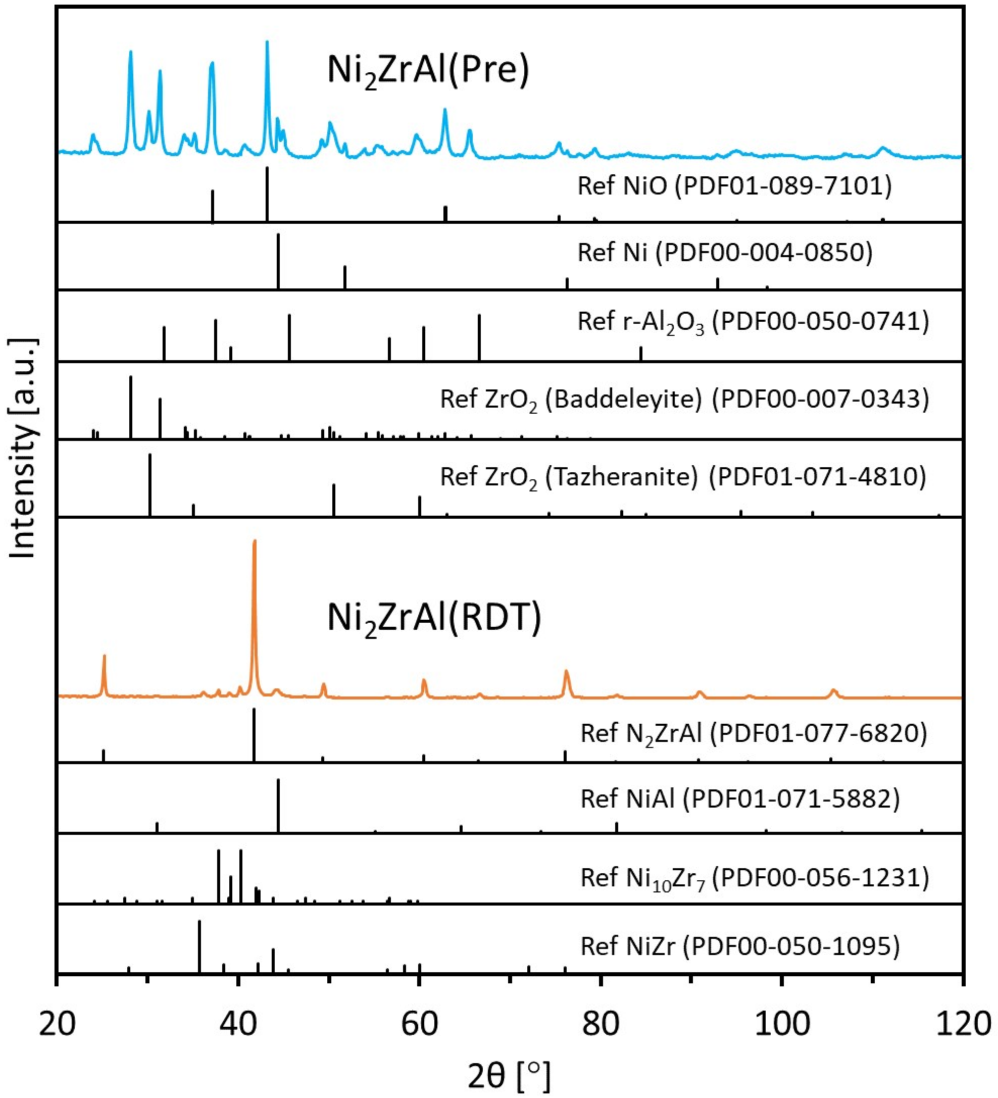

Fig. S9 XRD patterns of (a) an oxide precursor of Ni_2ZrAl(Pre) and (b) the reduced sample of Ni_2ZrAl(RDT) with possible references. For Ni_2ZrAl(Pre), the peaks were corresponding to metal oxides (NiO, γ-Al_2O_3, and ZrO_2) as well as metallic Ni. For Ni_2ZrAl(RDT), the peaks were mostly assigned to the targeted intermetallic phases. However, other than an intermetallic Ni_2ZrAl phase, small impurity phases were identified into NiAl, $Ni_{10}Zr_7$ and NiZr for Ni_2ZrAl(RDT), but they were negligible in comparison with Ni_2ZrAl. The crystallite size of Ni_2ZrAl was estimated as 34 nm from the Scherrer equation.

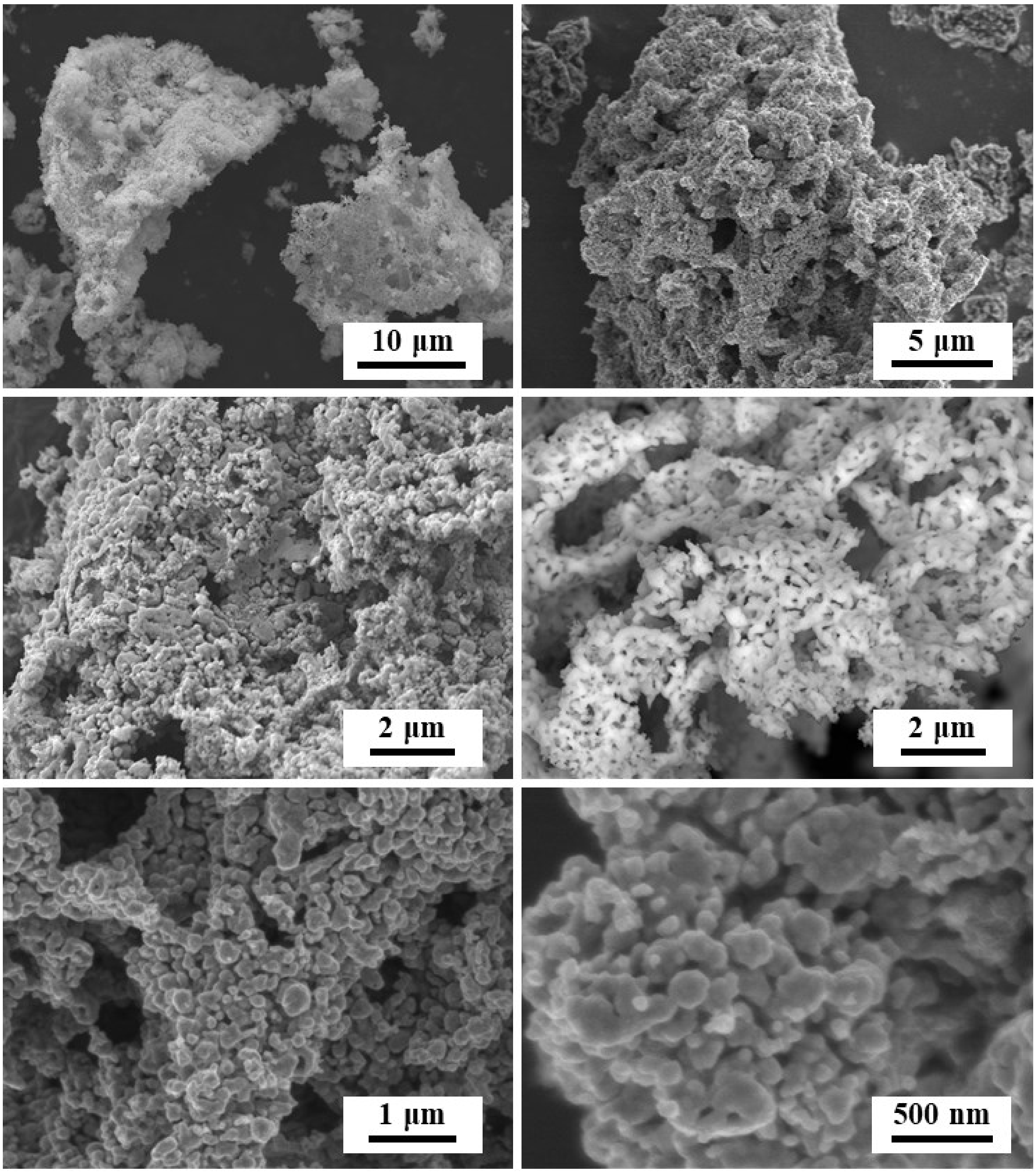

Fig. S10 SEM images of Ni_2ZrAl(RDT). Roughly estimating, the average particle size of Ni_2ZrAl(RDT) is larger than Ni_2TiAl(RDT). The XRD measurements also support the magnitude relationship.

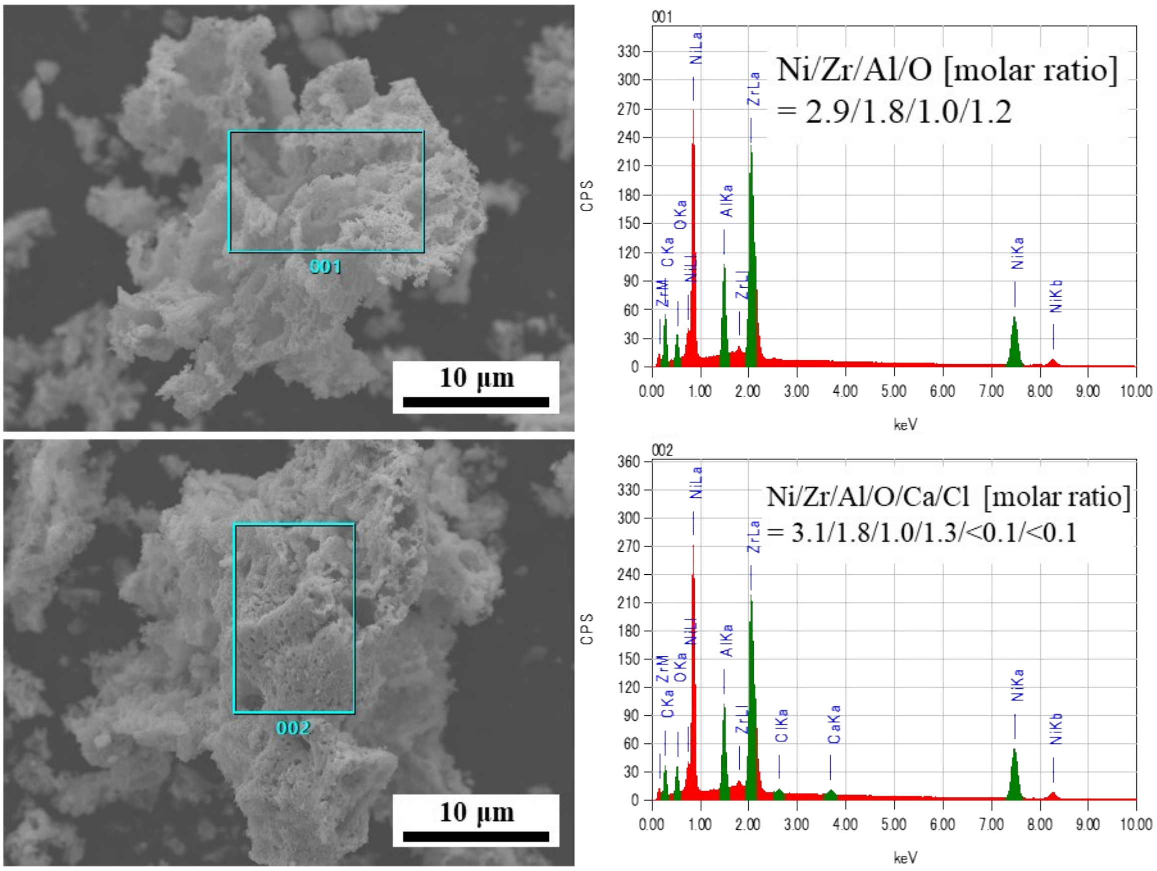

Fig. S11 SEM images and the elemental analysis of 2 different positions (001 and 002) on Ni_2ZrAl(RDT). The impurities, such as Ca and Cl, were little detected in the sample, indicating that the post-rinsing treatments by NH_4Cl solution effectively removed these element-related species.

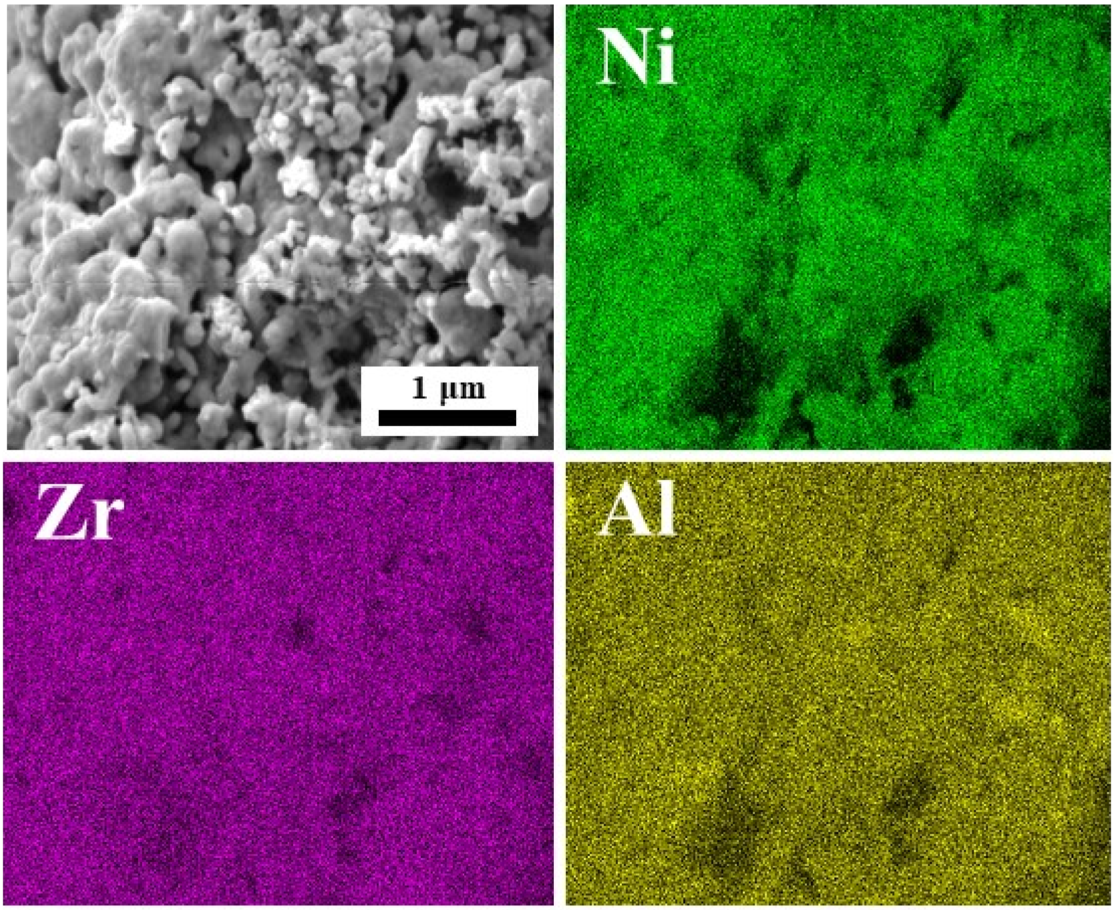

Fig. S12 A SEM image and the elemental mapping of Ni, Zr and Al on Ni_2ZrAl(RDT).

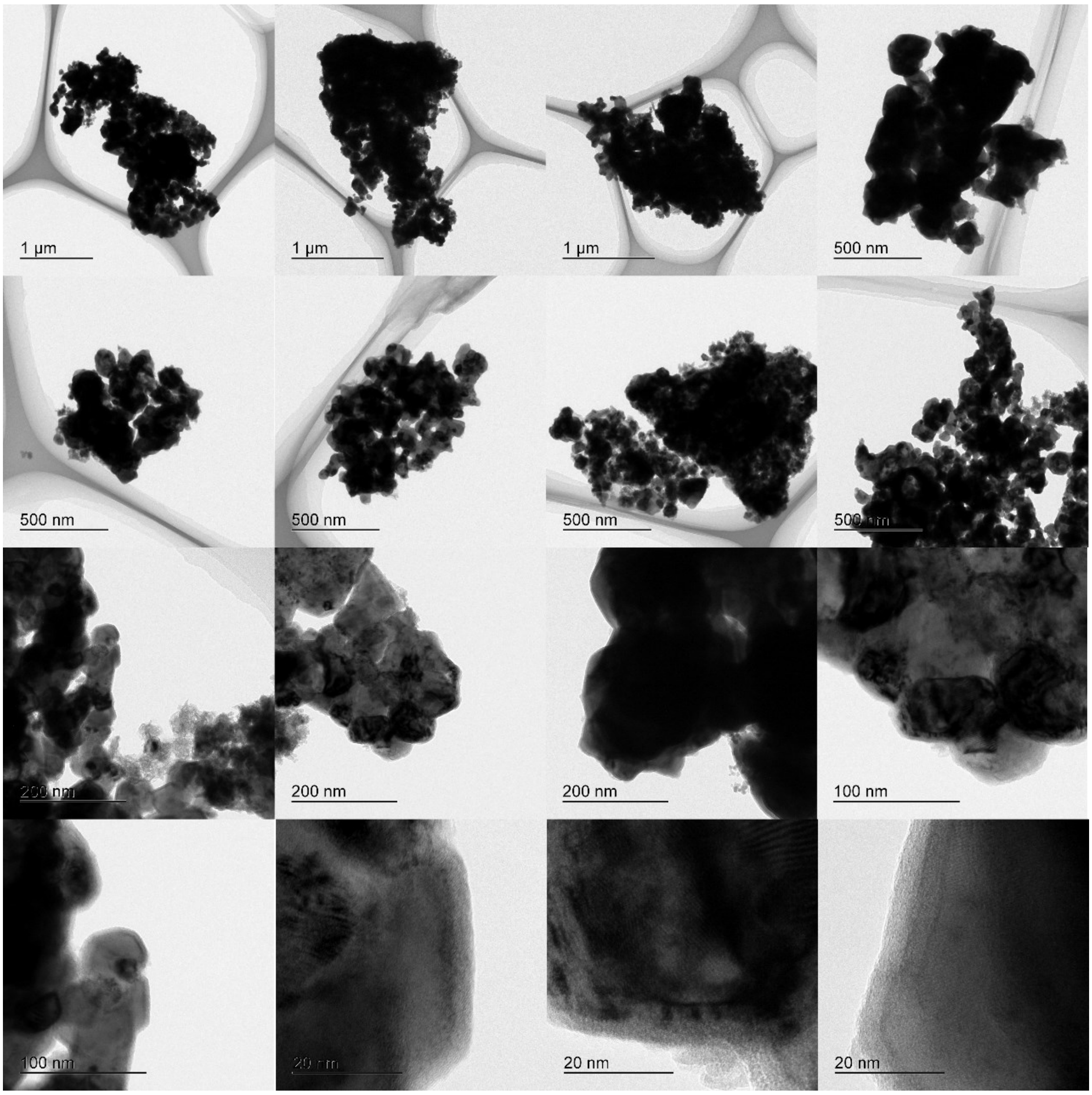

Fig. S13 TEM images of Ni_2ZrAl(RDT).

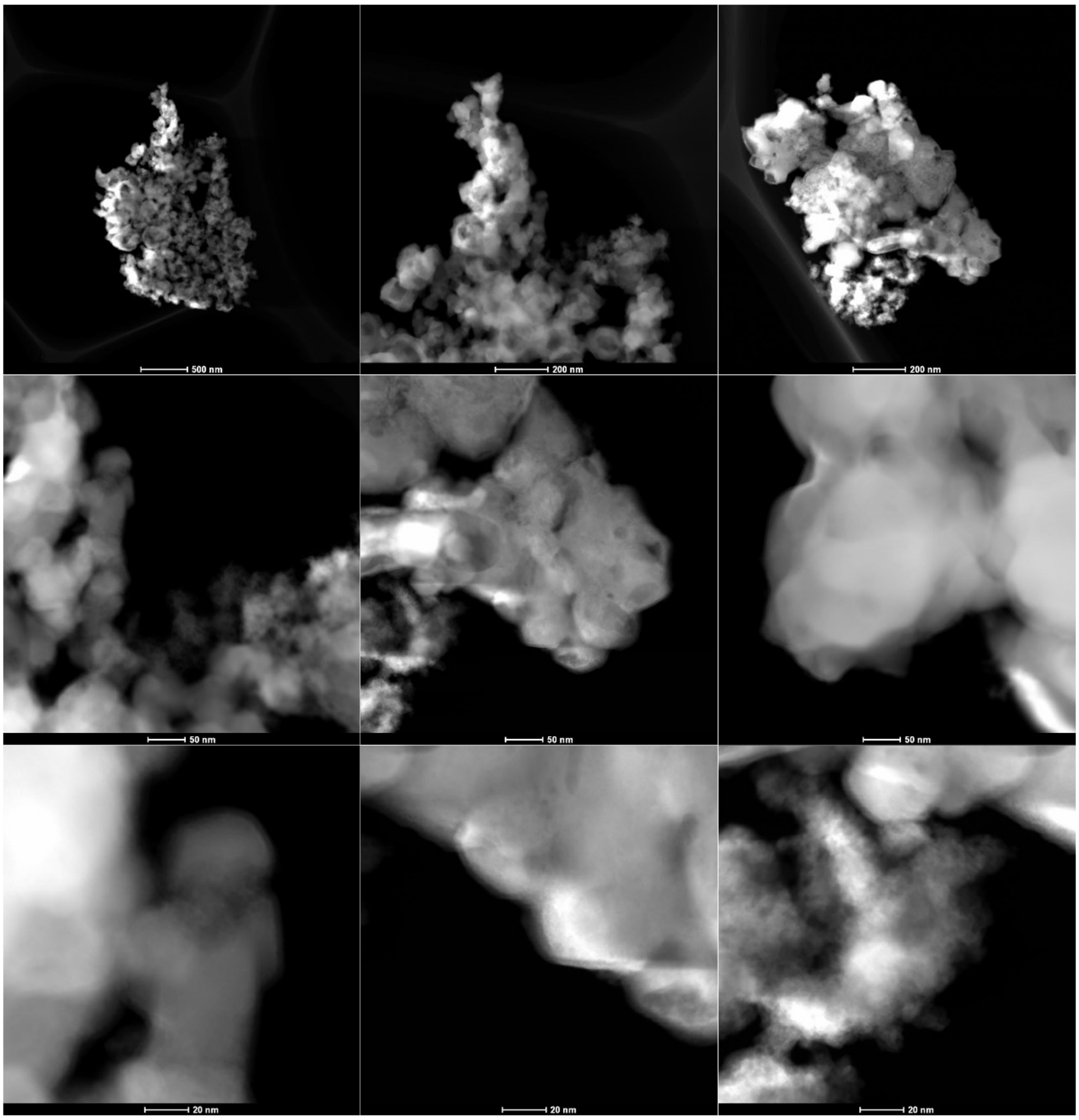

Fig. S14 HAADF-STEM images of Ni_2ZrAl(RDT).

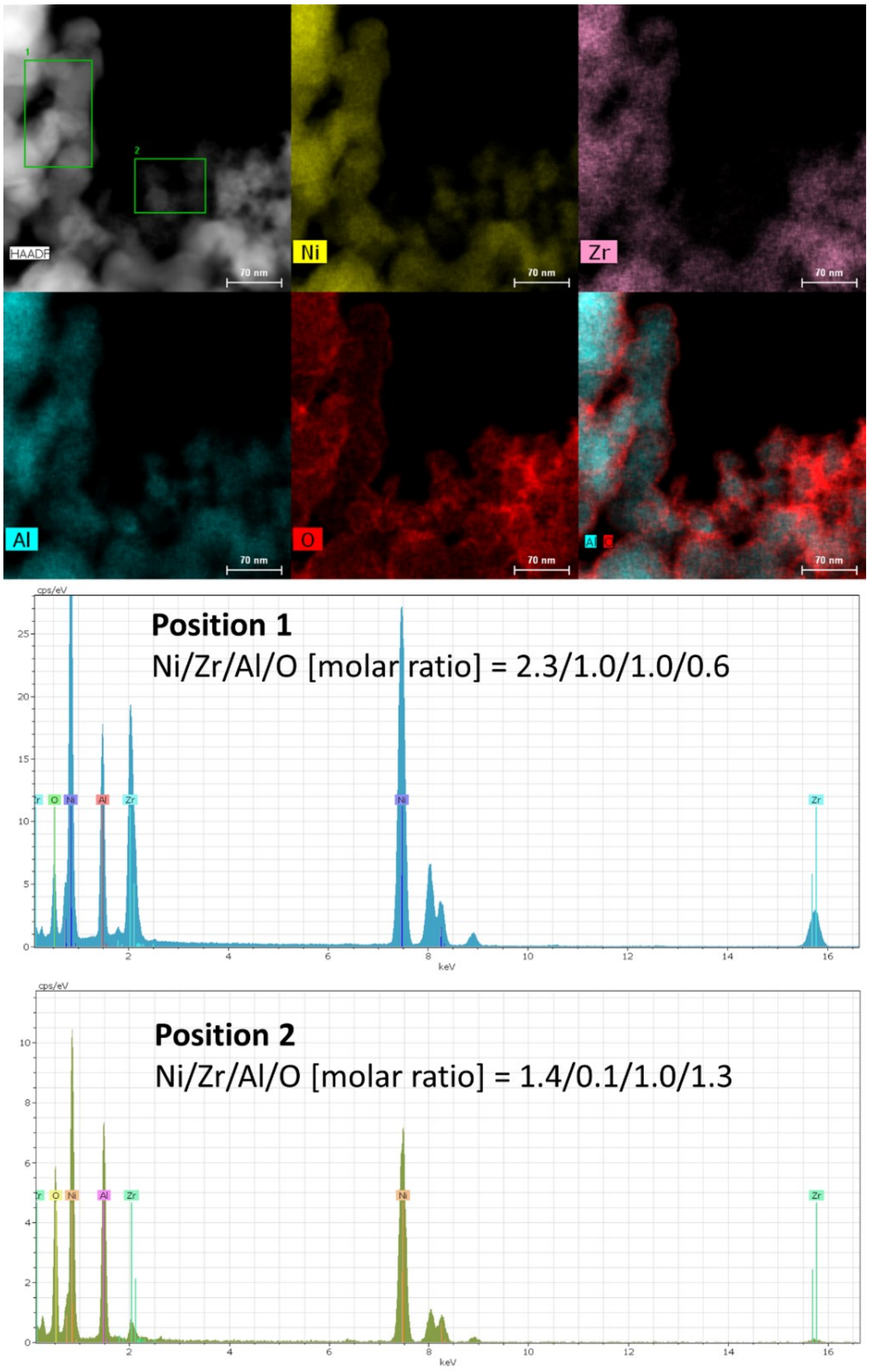

Fig. S15 HAADF-STEM images and elemental analysis of 2 different enclosed portions with molar ratios for Ni_2ZrAl(RDT).

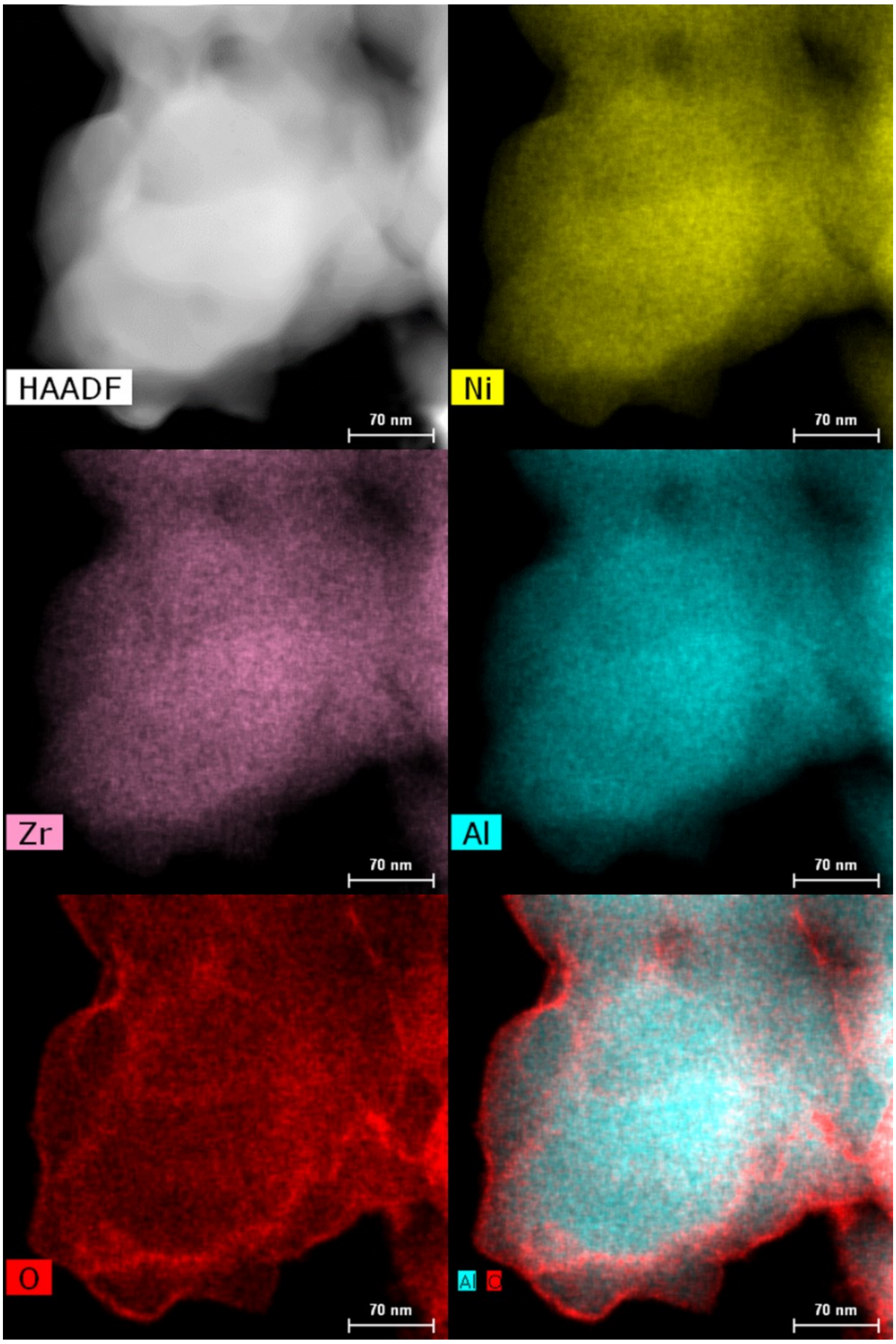

Fig. S16 HAADF-STEM images and elemental analysis for Ni_2ZrAl(RDT). We speculated that the activated surface is less stable from a thermodynamical point of view and more sensitive to oxygen than the bulk. Hence, the surface of the obtained sample could be partially oxidized to form stable oxide layers, such as ZrO_2, and Al_2O_3, with the thickness of a few nanometers that cannot be detected by XRD apparatuses.

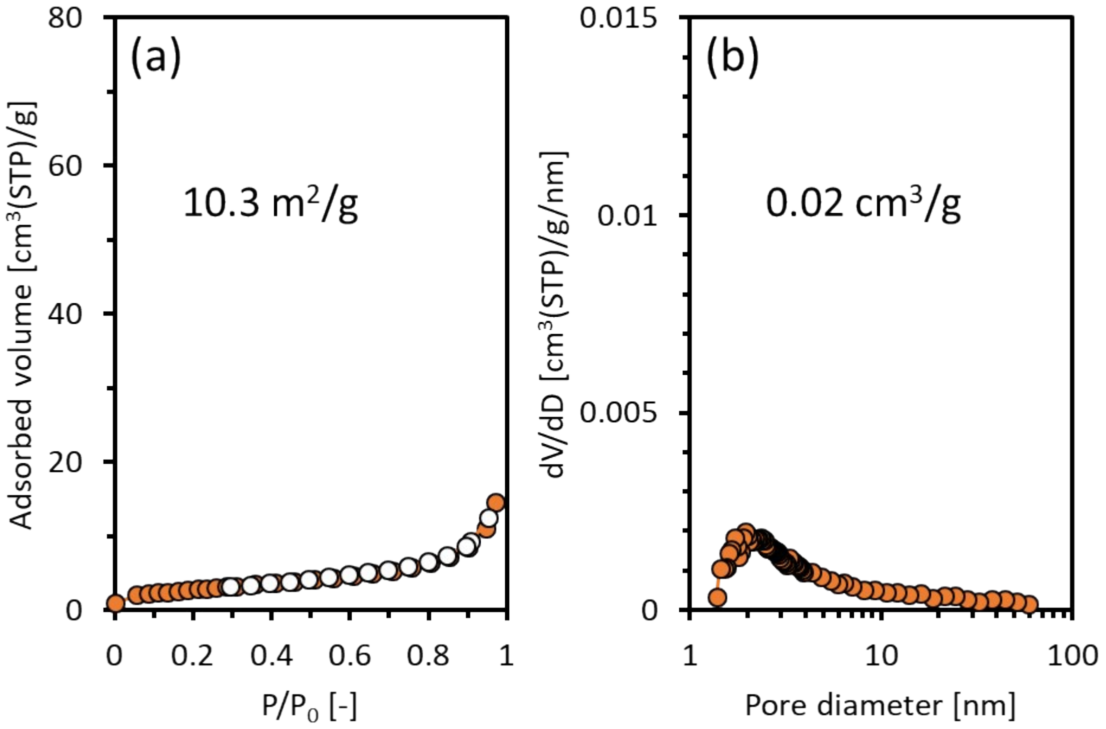

Fig. S17 (a) Adsorption and desorption isotherms of nitrogen and (b) the pore size distribution for Ni_2ZrAl(RDT).

Table S1 BET surface area (SA), pore volume (Vp), and particle size calculated by nitrogen adsorption and XRD measurement.

Sample	SA [m^2/g]	Vp [cm^3/g]	Particle size [nm]*
Ni_2TiAl(RDT)	70.8	0.11	24.0
Ni_2ZrAl(RDT)	10.3	0.02	34.4

* Calculated by the Sherrer equation with peaks observed at 43.4° and 41.7° for Ni_2TlAl and Ni_2ZrAl, respectively.

Table S2 Comparison of synthesis method and BET surface area (SA) between reported nickel-based aluminides.

Sample	Synthesis method	SA [m^2/g]	ref.
Ni_2TiAl(RDT)	Chemical route; CaH_2 reduction at 600 °C in a molten LiCl and rinsing at room temperature	70.8	This study
Ni_2ZrAl(RDT)		10.3	This study
NiAl		93.7-113.9	1, 2
Ni_3Al		13.1-27.3	2, 3
Ni_2TiAl	Physical route; Arc-melting and milling at room temperature	0.13	4-6
NiAl	Physical route; Hydrogen plasma-metal reaction under H_2-Ar flow	26.7	7
Ni_3Al		26.2	
NiAl	Chemical route; $LiAlH_4$ reduction at 164 °C in 1,3,5-trimethylbenzene and annealing at 650-700 °C	15	8
		26.9	9

Table S3 Number of active sites and BET surface area (SA) for relevant catalysts.

Catalyst	BET SA [m^2/g]	Number of sites per surface area [sites/m^2]	Number of sites per catalyst weight [sites/g]
NiAl	73.5	8.82×10^{18}	6.48×10^{20}
Ni_2TiAl(RDT)	70.8	7.13×10^{18}	5.05×10^{20}
Ni_2ZrAl(RDT)	10.3	6.32×10^{18}	6.51×10^{19}

The numbers of active sites were estimated from the BET surface area and atomic radii (Ni: 1.49 Å, Al: 1.18 Å, Ti: 1.76 Å, Zr: 2.06 Å).

Table S4 Comparison of TOF and Ea that were calculated by using CH_4 production rates and CO_2 reaction rates.

Catalyst	Reaction conditions for TOF calculation	TOF [x 100 s^{-1}]		Ea [kJ/mol]	ref.
		CH_4	CO_2		
NiAl	$CO_2/H_2/N_2$=1/4/5, GHSV=4200h^{-1}, 400℃	4.7	5.3	65	This study
Ni_2TiAl(RDT)		0.5	1.0	61	
Ni_2ZrAl(RDT)		4.6	11.2	104	
13Ni/Al_2O_3	CO_2/H_2=1/4, GHSV=36000h^{-1}, 410℃	-	82.0	-	10
5Ni/Al_2O_3	$CO_2/H_2/N_2$=60/15/20, F/W=2.5mL/g/s, 400℃	150	-	-	11
15Ni/Al_2O_3		191	-	-	
25Ni/Al_2O_3		202	-	-	
40Ni/Al_2O_3		132	-	-	
5Ni/TiO_2	CO_2/H_2/Ar=3/12/5, GHSV=1800-9000h^{-1}, 200℃	0.21	-	-	12
10%Ni/TiO_2		0.13	-	-	
15%Ni/TiO_2		0.12	-	-	
20%Ni/TiO_2		0.09	-	-	
4Ni/ZrO_2	CO_2/H_2=1/4, F/W=1.5mL/g/s, 200℃	43	-	-	13
Sponge Ni	$CO_2/H_2/N_2$=1/4/5, GHSV=4200h^{-1}, 250℃	-	-	70	14
Ni/CeO_2		-	-	99	

In comparison with TOFs, our catalysts give very low values than the common supported catalysts, such as Ni/Al_2O_3, Ni/TiO_2 and Ni/ZrO_2. As suggested by TEM-EDS analysis (**Fig. 3**, **Fig. S16**), the surface of used Ni_2TiAl and Ni_2ZrAl catalysts were covered by some oxides, such as Al_2O_3, TiO_2 and ZrO_2. It is impossible to reduce these oxides by a hydrogen pretreatment made at 500 °C. Therefore, the actual number of surface nickel site estimated from the BET surface areas on NiAl, Ni_2TiAl(RDT), and Ni_2ZrAl(RDT) could be much lower than the estimated values, possibly leading to the underestimation of TOF values in this study.

References

1. Y. Kobayashi, S. Tada and R. Kikuchi, *J. Chem. Eng. Jpn.*, DOI:10.1252/jcej.20we085, in press.
2. Y. Kobayashi, S. Tada and R. Kikuchi, *Chem. Lett.*, 2020, **49(4)**, 341.
3. Y. Kobayashi, *Chem. Lett.*, 2019, **48(12)**, 1496.
4. T. Kojima, S. Kameoka and A.-P. Tsai, *ACS Omega*, 2017, **2**, 147.
5. T. Kojima, S. Kameoka, S. Fujii, S. Ueda and A.-P. Tsai, *Sci. Adv.*, 2018, **4**, eaat6063.
6. T. Kojima, S. Kameoka and A.-P. Tsai, *ACS Omega*, 2019, **4**, 21666.
7. Z. Wang, A. L. Fan, W. H. Tian, Y. T. Wang and X. G. Li, *Mater. Lett.*, 2006, **60**, 2227.
8. X. Chen, Y. Ma, L. Wang, Z. Yang, S. Jin, L. Zhang and C. Liang, *ChemCatChem*, 2015, **7**, 978.
9. S. H. Kim, J. Choi, J. Yun and E.-W. Jeong, *RSC Adv.*, 2017, **7**, 16709.
10. David Méndez-Mateos, V. Laura Barrio, Jesús M. Requies and José F. Cambra, *RSC Adv.*, 2020, **10**, 16551.
11. Zhanming Zhang, Yue Tian, Lijun Zhang, Song Hu, Jun Xiang, Yi Wang, Leilei Xu, Qing Liu, Shu Zhang and Xun Hu, *Int. J. hydrog. Energy*, 2019, **44**, 9291.
12. Jie Liu, Changming Li, Fei Wang, Shan He, Hao Chen, Yufei Zhao, Min Wei, David G. Evans and Xue Duan, *Catal. Sci. Technol.,* 2013, **3**, 2627.
13. Michiaki Yamasaki, Hiroki Habazaki, Katsuhiko Asami, Kohichi Izumiya, Koji Hashimoto, *Catal. Comm.*, 2006, **7**, 24.
14. Shohei Tada, Shun Ikeda, Naohiro Shimoda, Tetsuo Honma, Makoto Takahashi, Akane Nariyuki, Shigeo Satokawa, *Int. J. hydrog. Energy*, 2017, **42**, 30126.
